# Supplementary material for: Dissociating frontoparietal brain networks with neuroadaptive Bayesian optimization
Source: Nat Commun. 2018 Mar 26;9:1227. doi: 10.1038/s41467-018-03657-3 (PMC5964320; doi:10.1038/s41467-018-03657-3)
Supplement: Supplementary file 1 — Supplementary Information(PDF 1891 kb) [file 41467_2018_3657_MOESM1_ESM.pdf]

Supplementary Information

Dissociating frontoparietal brain networks with neuroadaptive Bayesian optimization

Romy Lorenz, Ines R. Violante*, Ricardo Pio Monti*, Giovanni Montana, Adam Hampshire and Robert Leech

Overview

Supplementary Figure 1	Mean Euclidean distance between successive experimental conditions over time
Supplementary Figure 2	Subject-level Euclidean distance between successive tasks over time (Experiment 1)
Supplementary Figure 3	Subject-level results of Experiment 1
Supplementary Figure 4	BOLD activation across task space for dFPN and vFPN separately (Experiment 1)
Supplementary Figure 5	Subject-level results for Deductive Reasoning task (Experiment 2)
Supplementary Figure 6	Group-level Bayesian predictions across task parameter space for Tower of London task (Experiment 2)
Supplementary Figure 7	Subject-level results for Tower of London task (Experiment 2)
Supplementary Figure 8	Subject-level results of Experiment 3

Supplementary Methods	Brief description of other 14 tasks (Experiment 1 and Experiment 3)

Supplementary Results	Sampling behavior of acquisition function for alternative tasks (Experiment 1)

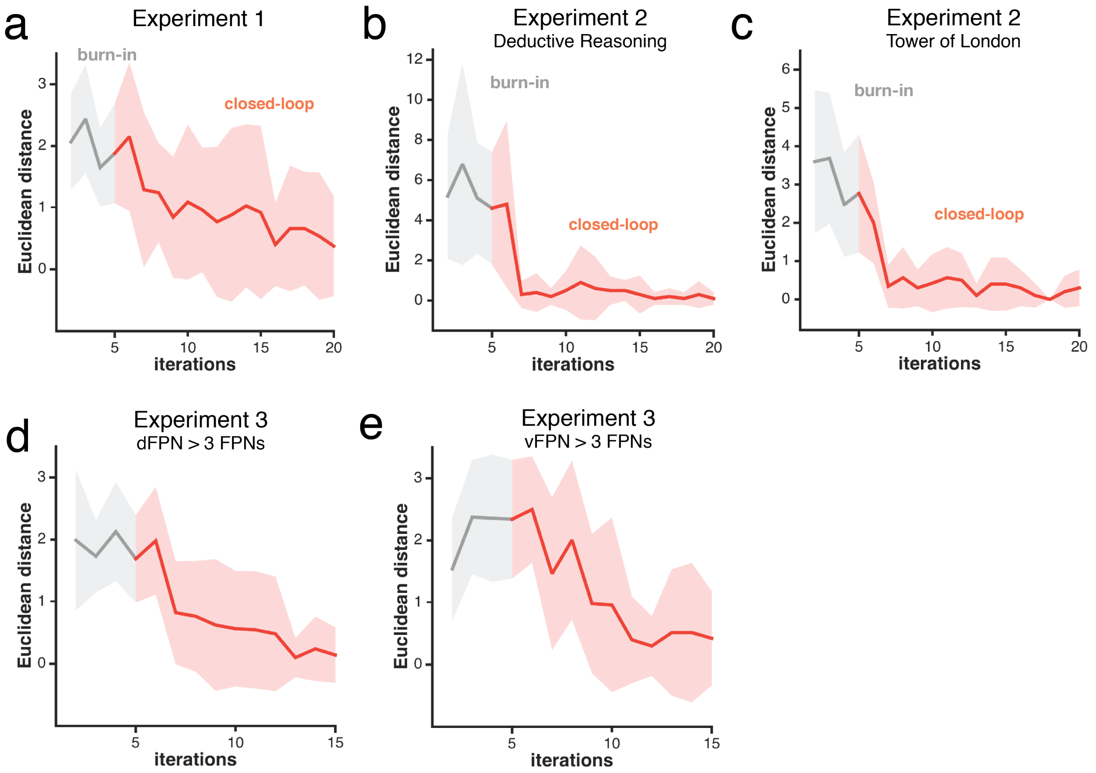

Supplementary Figure 1: Mean Euclidean distance between successive experimental conditions over time. Mean ± std (shaded areas) Euclidean Distance (ED) for **(a)** optimization across task space (Experiment 1), **(b)** optimization across task parameter space for Deductive Reasoning Task (Experiment 2), **(c)** optimization across task parameter space for Tower of London task (Experiment 2), **(d)** optimization across task space for the contrast dFPN > remaining three FPNs (Experiment 3) and **(e)** optimization across task space for the contrast vFPN > remaining three FPNs (Experiment 3). In order to obtain a first Bayesian model estimate, each optimization run was initiated with a burn-in period, i.e., five tasks were randomly selected from the task space and presented to the subject in the beginning of the experiment (iteration 1-5). This was followed by the closed-loop period (iteration 5-end), during which the acquisition function guided the search. In the case of successful optimization, the acquisition function transitions from exploration in the beginning of the run to exploitation later on (see Methods). Across all runs, we found a consistent decrease in ED over time, indicating that the algorithm selected experimental conditions further away in the task space in the beginning of the run (i.e. explorative phase), while it kept sampling the predicted optimal experimental condition or nearby, towards the end of the run (i.e., exploitative phase). This sampling behavior suggests successful optimization.

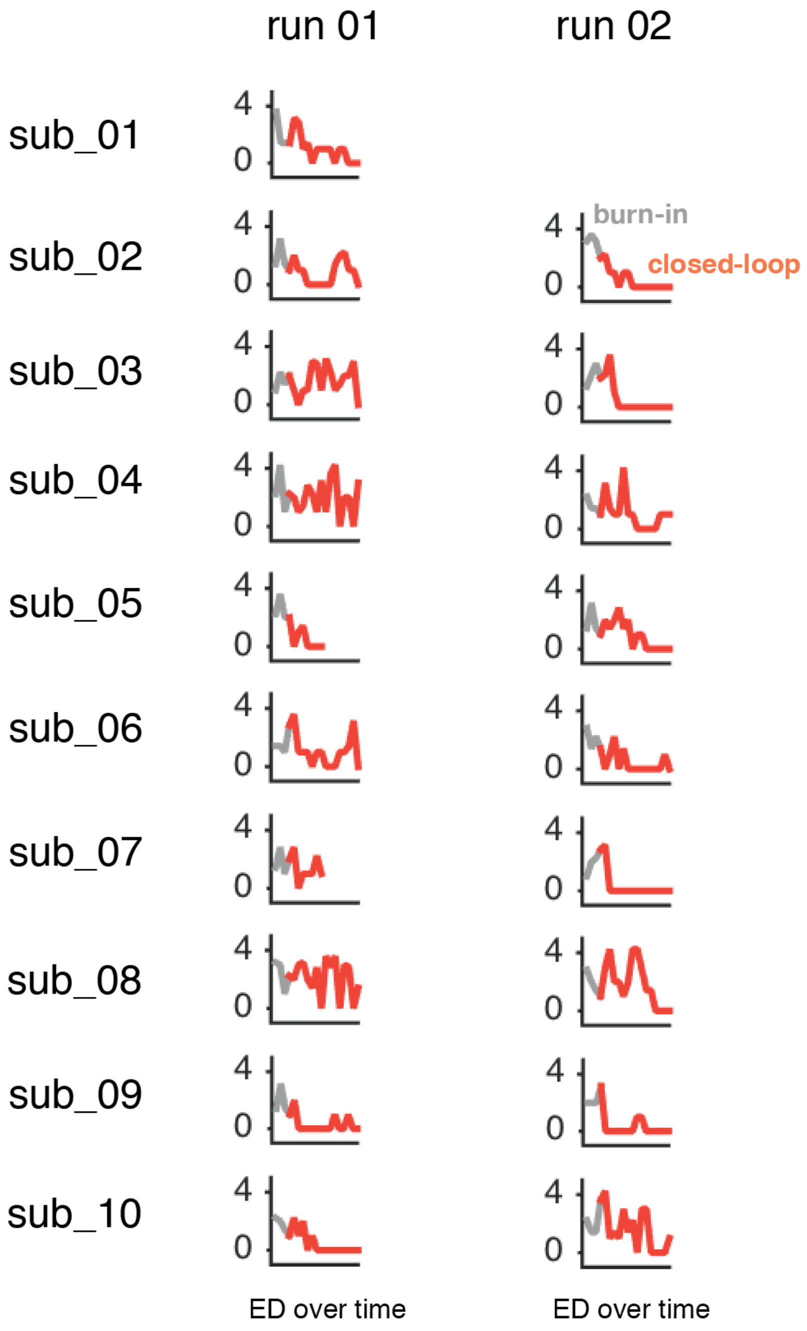

Supplementary Figure 2: Subject-level Euclidean distance between successive tasks over time (Experiment 1). Euclidean Distance (ED) for every subject (row) and each run (column) separately show that for the majority of runs the scanning time could have been reduced by several task blocks; however, for others a longer optimization period could potentially have resulted in more stable results. This highlights the need for developing online stopping criteria in the future that automatically end the run only when the uncertainty of the algorithm over the experiment space is sufficiently small and/or enough statistical evidence has been accumulated. For sub_01 only one run was conducted; for two subjects (sub_05 and sub_07), one out of the two runs stopped after only 13 blocks due to technical failure.

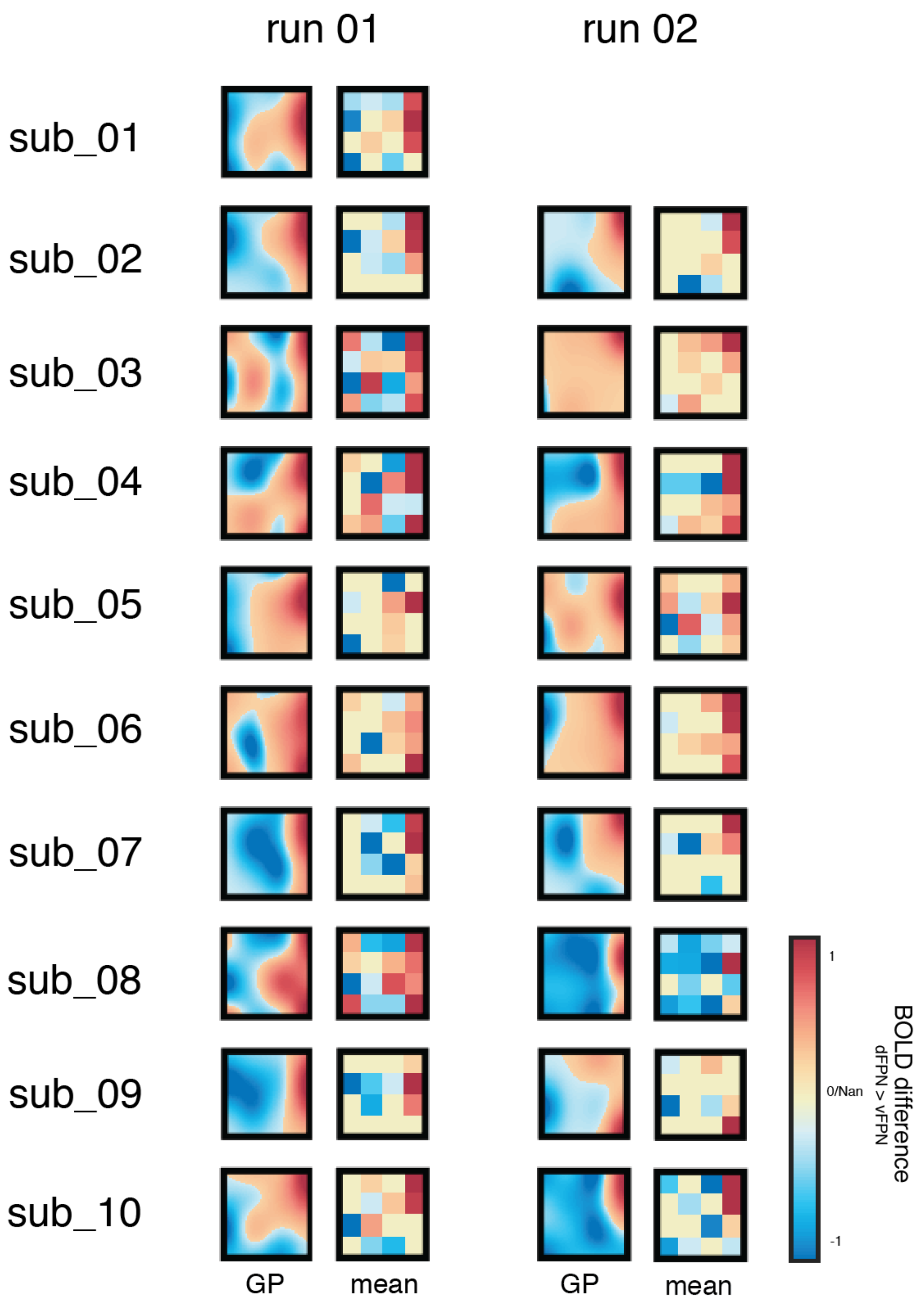

Supplementary Figure 3: **Subject-level results of Experiment 1.** Our result that the Tower of London and Deductive Reasoning task were optimal for dissociating the two FPNs was consistent within (column) and across subjects (row) as can be seen from the Bayesian prediction across the whole task space ('GP' panels). Bayesian predictions are based on all available observations from a single run (for sub_01 only one run was conducted) with fixed hyper-parameters (same as in real-time setting). To explore if the GP regression resulted in sensible predictions, we also plotted the mean value for each cell of the 4x4 task space ('mean' panels). For visualization purposes, for each plot, we re-scaled all positive contrast values between 0.2 and 1 and all negative contrast values between -1 and -0.2. This procedure had the benefit of keeping important information about the "sign" of the contrast values while facilitating plotting using an identical scaling throughout all subplots. Additionally, this facilitates inspecting the sampling behavior of the acquisition function in the 'mean' panels, i.e., light yellow corresponds to points where the acquisition function did not sample (i.e., NaN) while the Bayesian model (i.e., GP regression) extrapolates its predictions also to unseen points within the task space ('GP' panel).

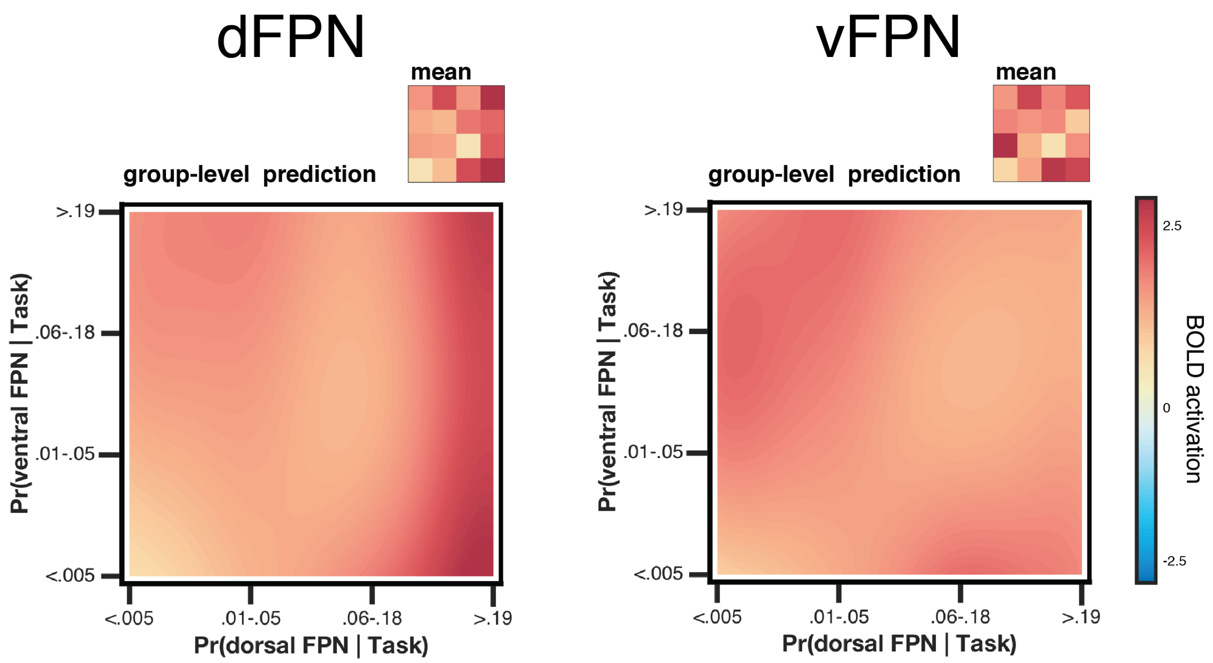

Supplementary Figure 4: BOLD activation across task space for dFPN and vFPN separately (Experiment 1). Instead of only presenting group-level predictions (i.e., GP regression) based on the contrast between dFPN and vFPN, here we repeat the same analysis but using the BOLD activation values for the dFPN and vFPN, separately. To explore if the regression resulted in sensible predictions, we also plotted the mean value across all subjects for each cell of the task space ('mean' panels). We found that the two identified tasks in Experiment 1 (i.e., Deductive Reasoning and Tower of London tasks) were not selected because they induced a negative activation in the vFPN, but instead just lower activation.

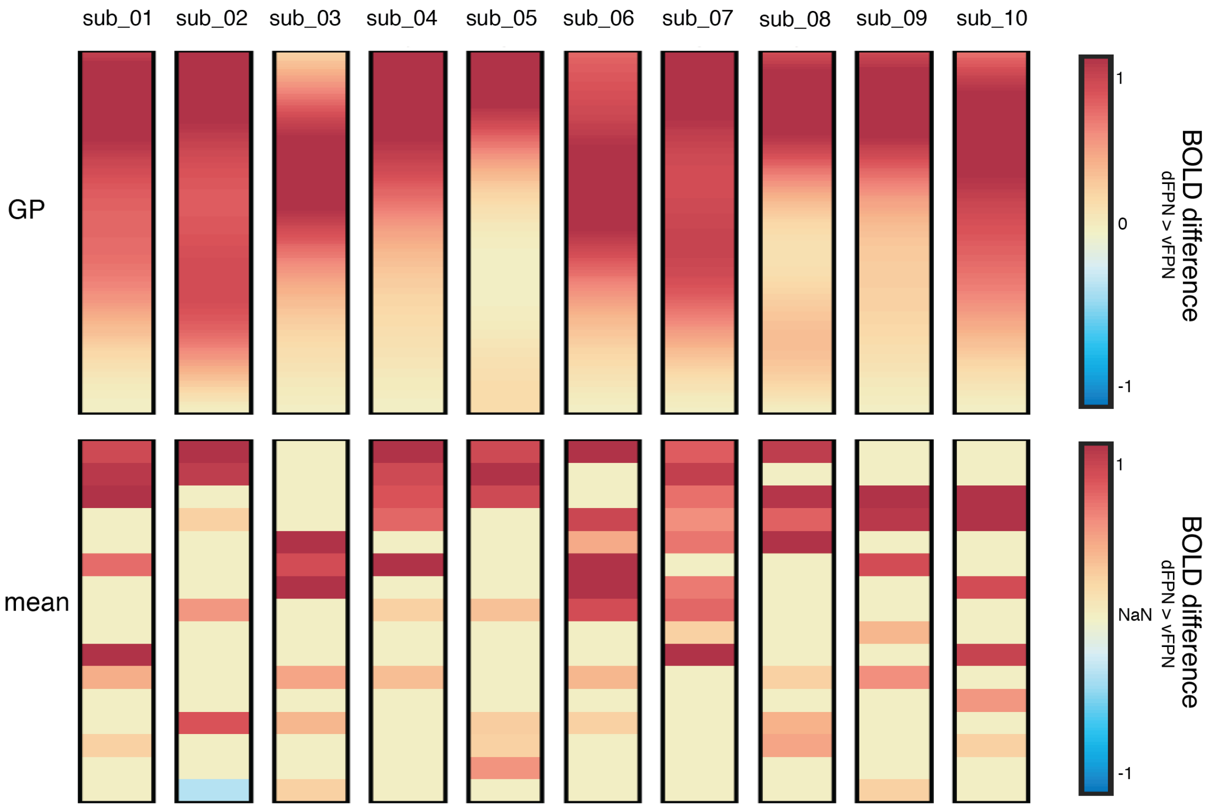

Supplementary Figure 5: Subject-level results for Deductive Reasoning task (Experiment 2). Results for the Deductive Reasoning task were consistent across subjects as can be seen from the Bayesian prediction across the whole task space ('GP' panels). Bayesian predictions are based on all available observations from a single run with fixed hyper-parameters (same as in real-time setting). To explore if the GP regression resulted in sensible predictions, we also plotted the mean value for each cell of the 16x1 task parameter space ('mean' panels). For visualization purposes, for each plot, we re-scaled all positive contrast values between 0.2 and 1 and all negative contrast values between -1 and -0.2. This procedure had the benefit of keeping important information about the "sign" of the contrast values while facilitating plotting using an identical scaling throughout all subplots. Additionally, this facilitates inspecting the sampling behavior of the acquisition function in the 'mean' panels, i.e., light yellow corresponds to points where the acquisition function did not sample (i.e., NaN) while the Bayesian model (i.e., GP regression) extrapolates its predictions also to unseen points within the task parameter space ('GP' panel).

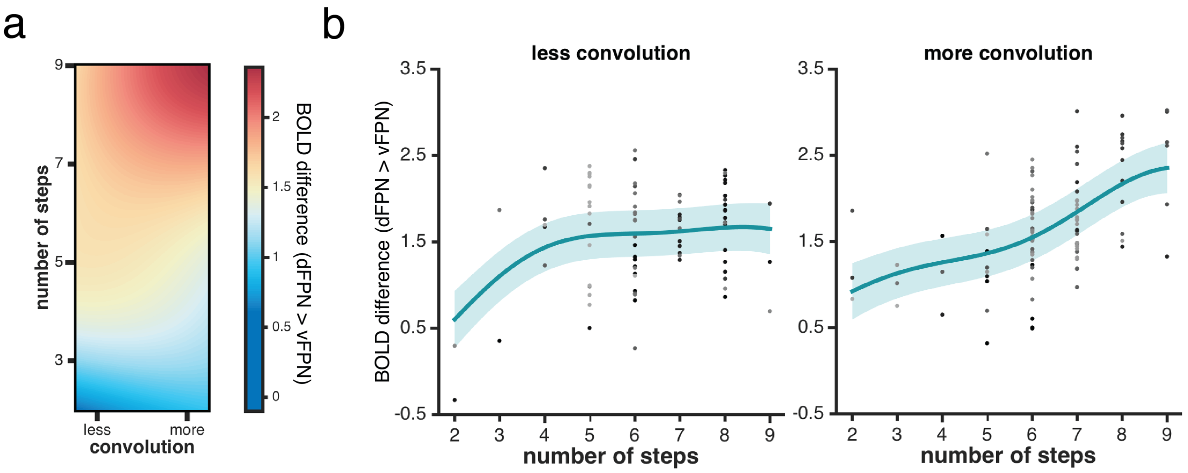

Supplementary Figure 6: Group-level Bayesian predictions across task parameter space for Tower of London task (Experiment 2). (a) Mean group-level predictions. **(b)** Mean ± std (shaded areas) group-level predictions sliced through the convolution dimension. Each dot represents one observation and observations are colored (grayscale intensity) by subject. Results suggest distinct predictive patterns across the convolution dimension; however, these effects seem to be driven by a few individuals. This assumption was confirmed when conducting a LME analysis that showed a significant quadratic effect of number of steps with brain activity but no significant convolution or interaction effect (reported in Results section, also see Supplementary Fig. 5).

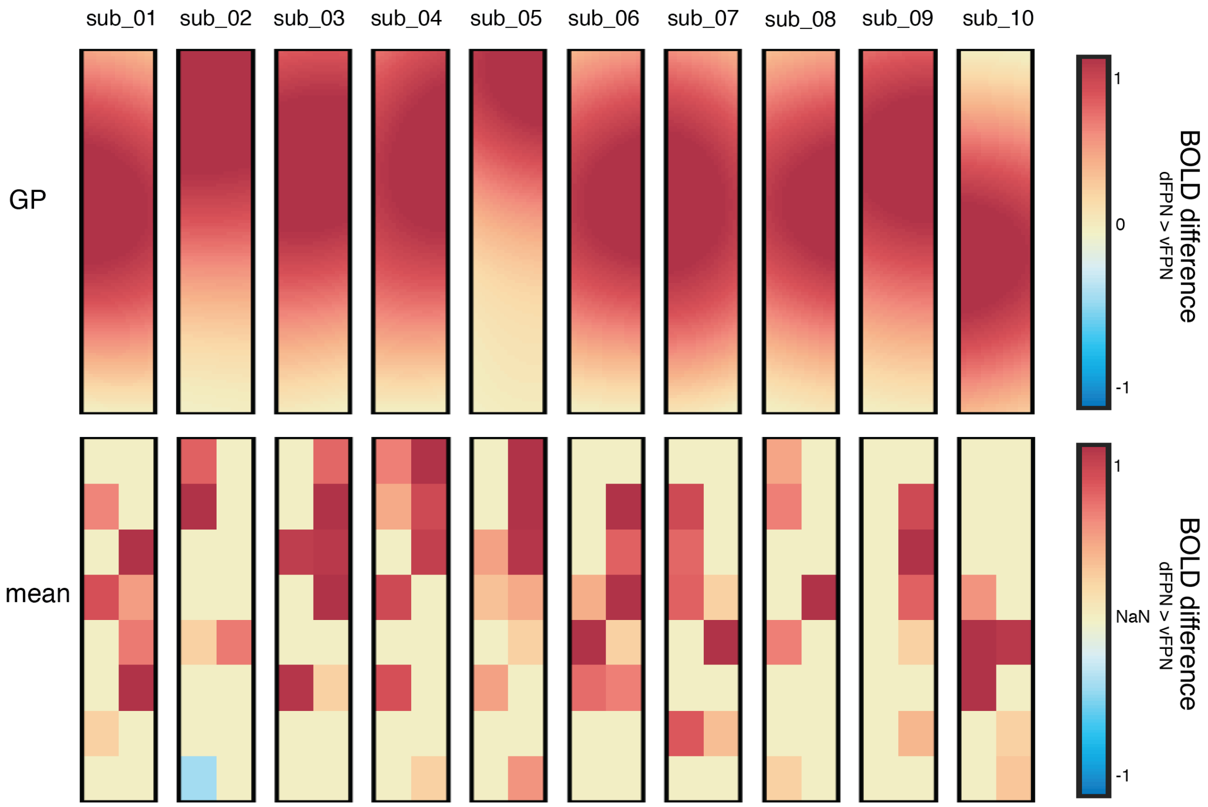

Supplementary Figure 7: Subject-level results for Tower of London task (Experiment 2). Results for the Tower of London task were consistent across subjects as can be seen from the Bayesian prediction across the whole task space ('GP' panels). Bayesian predictions are based on all available observations from a single run with fixed hyper-parameters (same as in real-time setting). To explore if the GP regression resulted in sensible predictions, we also plotted the mean value for each cell of the 8x2 task parameter space ('mean' panels). For visualization purposes, for each plot, we re-scaled all positive contrast values between 0.2 and 1 and all negative contrast values between -1 and -0.2. This procedure had the benefit of keeping important information about the "sign" of the contrast values while facilitating plotting using an identical scaling throughout all subplots. Additionally, this facilitates inspecting the sampling behavior of the acquisition function in the 'mean' panels, i.e., light yellow corresponds to points where the acquisition function did not sample (i.e., NaN) while the Bayesian model (i.e., GP regression) extrapolates its predictions also to unseen points within the task parameter space ('GP' panel).

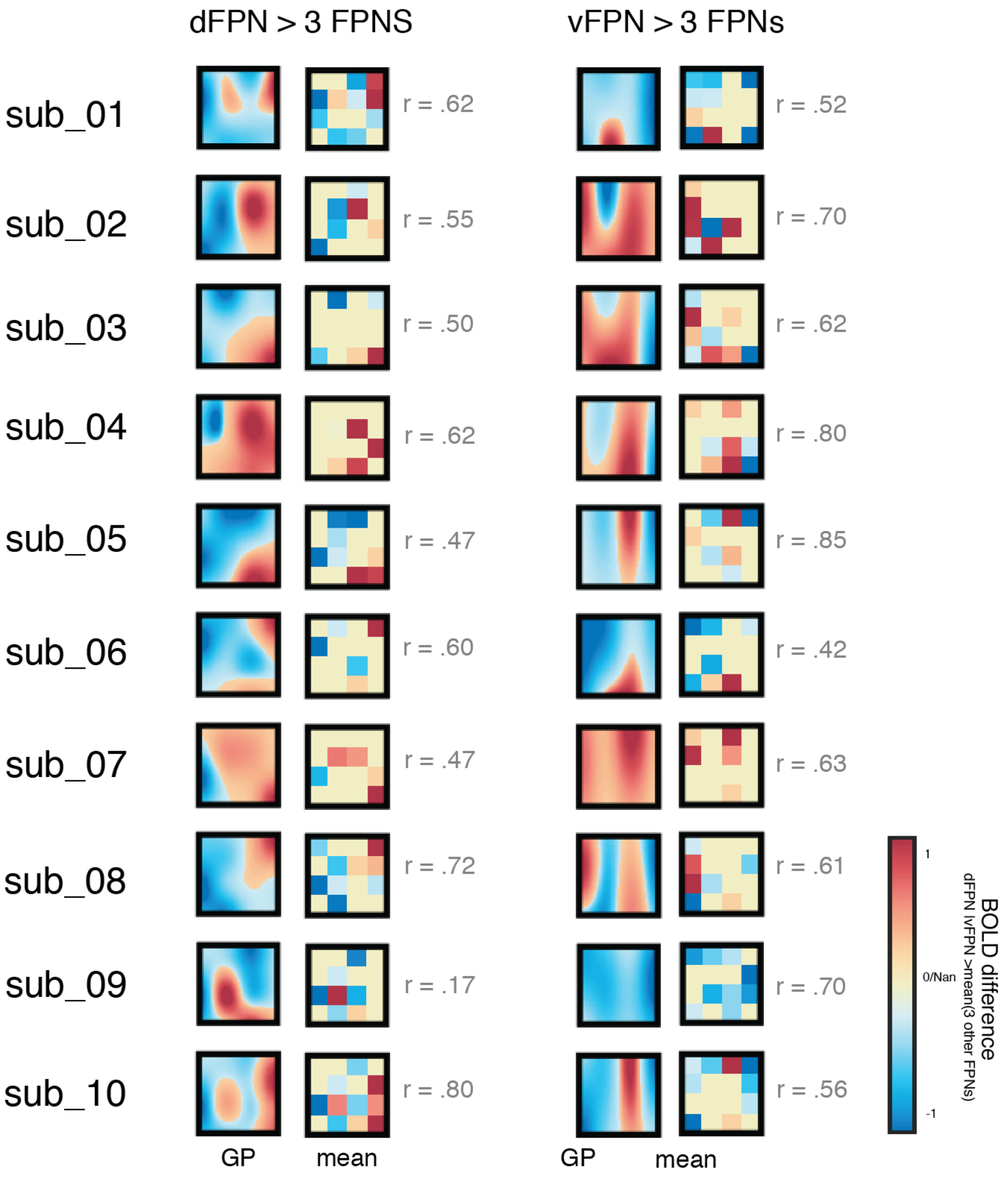

Supplementary Figure 8: Subject-level results of Experiment 3. Bayesian predictions ('GP' panels) are based on all available observations from a single run with fixed hyper-parameters (same as in real-time setting). To explore if the GP regression resulted in sensible predictions, we also plotted the mean value for each cell of the 4x4 task space ('mean' panels). We performed an additional analysis comparing group-level Bayesian predictions (Fig. 5b/5d) with subject-level Bayesian predictions ('GP' panels) using Spearman correlation; the results of this analysis are provided here next to the 'mean' panels in grey (r = .XY). Based on these results, we see that individuals' results moderately to highly correlate with the group effects, and that the group results are not driven by a small subset of participants. For visualization purposes, for each plot, we re-scaled all positive contrast values between 0.2 and 1 and all negative contrast values between -1 and -0.2. This procedure had the benefit of keeping important information about the "sign" of the contrast values while facilitating plotting using an identical scaling throughout all subplots. Additionally, this facilitates inspecting the sampling behavior of the acquisition function in the 'mean' panels, i.e., light yellow corresponds to points where the acquisition function did not sample (i.e., NaN) while the Bayesian model (i.e., GP regression) extrapolates its predictions also to unseen points within the task space ('GP' panel).

Supplementary Methods: Brief description of other 14 tasks (Experiment 1 and Experiment 3)

All tasks were presented in blocks for 35 s followed by 19 s of rest (black background). Preceding each task, participants received a brief instruction (5 s) about the task they would need to perform in the upcoming block followed by a short rest period (3 s). For task descriptions of the Tower of London Task as well as the Deductive Reasoning Task, please refer to the Methods section of the paper.

Posner Task | A white cross was presented in the center of the screen throughout the task block. For each trial, a filled white rectangle was presented for 0.25 s either to the left or right side of the cross. Subjects were instructed to respond as quickly as possible to this stimulus by pressing either the left or right button depending on the side of appearance. For 80% of the trials, the appearance of the filled white rectangle was spatially cued 0.1 s before by a rapid presentation of an unfilled white rectangle. In half of these cases, the cue was either congruent (i.e., on the same side as where the filled white rectangle was about to appear) or incongruent (i.e., on the opposite side as where the filled white rectangle was about to appear). The inter-trial interval randomly varied between 1.5 and 2.5 s; subjects performed between 13 and 14 trials per task block.

Anti-Saccade Task | A white cross was presented in the center of the screen throughout the task block. When no stimulus was present, subjects were instructed to fixate the cross. For each trial, a filled white rectangle appeared for 0.25 somewhere in the periphery of the screen. Subjects were instructed to make a saccade in the direction away from the white rectangle. After performing this “anti-saccade”, they were instructed to return to the fixation cross. The inter-trial interval randomly varied between 2 and 3 s; subjects performed between 12 and 16 trials per task block.

Go-No Go Task | For each of the 36 trials (i.e., every 0.7 s), a blue filled square was presented for 0.25 s in the center of the screen. Subjects were instructed to press the right button as quickly as possible every time the blue square appeared. In 8 trials (i.e., 20% of cases), instead of the blue square, a blue cross was presented, for which subjects were instructed to withhold their button press response.

Imagined Movement Task | For each of the 8 trials an arrow either pointing to the left or right appeared in the center of the screen for 4s. Subjects were instructed to imagine a left or right hand movement depending on the direction of the arrow. For imagined movement, subject were instructed perform first person proprioceptive imagery, rather than third person or visual imagery. After each trial a fixation cross in the center of the trial was shown for 0.4 s.

Oddball Discrimination Task | For each of 36 trials (every 0.7 s), a green plus was presented for 0.25 s in the center of the screen. However, in 8 trials (i.e., 20% of the cases), instead of a green plus sign, a green minus sign was presented as the “odd stimulus”, for which subjects were instructed to respond as quickly as possible by pressing the right button.

Encoding Task | For each of the four trials, subjects were presented with two sequences consisting of five digits ranging between 0 and 9. Subjects were instructed to memorize the first sequence and indicate if the second sequence was a

match or mismatch with the first sequence. The beginning of each sequence was indicated by showing the words “1. sequence” or “2. sequence” for 1 s in the center of the screen. Within each sequence, each digit was presented sequentially for 0.4 s in the center of the screen. After the first sequence was shown, the second sequence commenced. In the case of a mismatch, the second sequence only differed on one of the five digits from the first sequence. At the end of the second sequence, the question “Was this sequence a match?” was shown for 2 s and subjects indicated their response by either pressing the left button for “Match” or right button for “Mismatch”.

Reading Overt Task | Subjects were presented with two excerpts from “The Little Prince”. Subjects were instructed to read the text aloud while avoiding any head movement.

Theory of Mind Task | For each of the three trials, subjects were presented with a short story that was presented for 9 s. The story was followed by a brief description of a belief state of one of the persons in that story, which was presented for 5 s. Subjects were instructed to indicate if they think the belief state of the person was true or false by either pressing the left or right button, respectively. After each trial, a white cross in the center for the screen was presented for 1 s. Stories were used from a previous study[1].

Fixation Cross | A white cross was presented in the center of the screen that subjects were instructed to fixate on for the duration of the task block.

Counting / Calculation Task | For each trial, subjects were presented with a summation problem. Each summation problem displayed the sum of two numbers between 1 and 20, which was correct in 50% of all trials. Subjects were instructed to indicate if the presented sum was correct or incorrect by pressing the right or left button, respectively. The inter-trial interval randomly varied between 1.7 and 3.5 s; subjects performed between 12 and 14 trials per task block.

Flashing Checkerboard Task | Subjects were instructed to passively watch a checkerboard flashing at a frequency of 2 Hz while fixating on the red dot in the center of the screen.

Passive Listening Task | Subjects were instructed to passively listen to a 35 s music excerpt. The excerpt was from a song by ambient artist Robert Rich.

Divided Auditory Attention Task | For each of the 36 trials (i.e., every 0.7 s), a visual and auditory stimulus were presented simultaneously. The visual stimulus was presented for 0.25 s in the center of the screen. In 28 trials (i.e., 80% of the cases), the visual stimulus was a green plus sign. However, in 8 trials (i.e., 20% of the cases), a green minus sign was presented as the “odd visual stimulus”, for which subjects were instructed to respond to as quickly as possible by pressing the right button. Similarly, for 28 trials, the auditory stimulus was a low-pitched beep while in 8 trials a high-pitched beep was presented as the “odd auditory stimulus”, for which subjects were instructed to respond as quickly as possible by pressing the left button. The appearance of the “odd visual” and “odd auditory” stimuli was selected at random, independently from each other.

Wisconsin Card Sorting Task | For each trial, three shape stimuli were presented to the subject. The cards differed in the color, number and form of the shapes. The subject was instructed to pair one of the two cards from the top row to the "target card" in the bottom row. The subject was not instructed what stimulus dimensions to use for pairing the cards, but received immediate feedback in the form of "Good", if a particular pairing was correct, or "Wrong" if a pairing was incorrect. After at least 5 subsequent trials with the same pairing order, the pairing rules were changed and the participant needed to find out the new pairing rule in order to be successful. After each trial, a white fixation cross in the center of the screen was presented for 0.5 s. This task was self-paced as it depended on the subject's button press in order to give feedback; therefore, each subject had a different number of trials (range: 8-17, median: 13) per task block.

Supplementary Results: Sampling behavior of acquisition function for alternative tasks (Experiment 1)

One possible concern is that the result of the predicted optimum for the Tower of London task and Deductive Reasoning task may be driven by the acquisition function over-exploiting this corner of the task space due to the particular arrangement of the cognitive tasks in that space. In such a case, higher difference between the two FPNs for the Go/No-Go task or the Encoding task in contrast to the Fixation Cross or the Divided Auditory Attention task might impede sufficient exploration of the part of the experiment space where the Wisconsin Card Sorting and Counting/Calculation tasks are placed – due to the smoothness assumptions encoded in our Bayesian model (i.e., the prior belief that experimental conditions close to each other elicit a similar brain responses). In order to assess this alternative explanation, we computed for how many subjects and runs either the Wisconsin Card Sorting or the Counting/Calculation task have been sampled at least once. We found that in at least one run for all 10 subjects, or 14 out of 19 runs in total, the two tasks were sampled on average 2.08 ± 2.86 (mean ± std) times. Therefore, the results obtained in Stage 2 could not be explained by insufficient exploration of these tasks.

Supplementary References

1. Dodell-Feder, D., Koster-Hale, J., Bedny, M., & Saxe, R. (2011). fMRI item analysis in a theory of mind task. *NeuroImage*, *55*(2), 705–712. https://doi.org/10.1016/j.neuroimage.2010.12.040
